# A Call for Use of Lipid Fractionation Studies in Patients With Abnormal Standard Lipid Profiles

**DOI:** 10.1210/jcemcr/luaf117

**Published:** 2025-07-10

**Authors:** Megan Kawamura, Isabel Casimiro

**Affiliations:** Department of Medicine, University of Hawaii John A. Burns School of Medicine, Honolulu, HI 96813, USA; Department of Medicine, University of Hawaii John A. Burns School of Medicine, Honolulu, HI 96813, USA; Department of Medicine (Endocrinology), Queens University Medical Group, Honolulu, HI 96813, USA

**Keywords:** lipid fractionation, hyperlipidemia, cardio IQ, PCSK9-inhibitors, bempedeoic acid, small LDL

## Abstract

A 54-year-old female individual with a history of hyperlipidemia, hypothyroidism, latent autoimmune diabetes of the adult (LADA) with hypoglycemia unawareness, vitiligo, and asthma presented with exercise-limiting pleuritic chest pain and dyspnea. She underwent a full pulmonary and cardiac workup locally and at Mayo Clinic, which were normal and negative for pulmonary embolism and showed no evidence of cardiac ischemia. Her family history and abnormal lipid panels suggested possible familial hypercholesterolemia by Dutch criteria. Lipid fractionation studies were done to further assess abnormal lipid levels and symptoms, as she lives a generally healthy and active lifestyle. Lipid fractionation studies revealed abnormal small, dense (sdLDL) and low-density lipoproteins (LDL) particle numbers. She was started on a triple cholesterol management regimen of ezetimibe, evolocumab, and bempedoic acid, which improved lipid fractionation values and symptoms. This case highlights the utility of using lipid fractionation studies in addition to traditional lipid panels to best treat patients with abnormal lipid panels on lipid-lowering therapy.

## Introduction

Monitoring of lipid levels is essential for managing hypercholesterolemia and preventing cardiovascular disease (CVD). While strong evidence shows that lowering low-density lipoprotein cholesterol (LDL-C) reduces CVD risk, hypercholesterolemia and CVD remain highly prevalent. Research on LDL particle composition and count suggests that small, dense LDL (sdLDL) and high LDL particles are linked to an increased risk of premature coronary artery disease (CAD). Increased levels of sdLDL contribute to atherogenesis due to its prolonged circulation time, ease of oxidization, proteoglycan binding, and endothelial penetration [1]. As a result, growing epidemiological evidence supports the use of lipid fractionation assays—specifically measuring sdLDL particle numbers—as valuable clinical tools for optimizing hypercholesterolemia management and reducing CVD risk.

Lipoprotein fractionation tests measure levels and characteristics of different types of lipoprotein particles such as lipoprotein (a) [Lp(a)], as well as high-density lipoprotein (HDL) subclasses which can be used to assess an individual's cardiovascular disease risk and potentially guide treatment. Three main lipid fractionation methods include (i) nuclear magnetic resonance (NMR), such as the NMR LipoProfile® (Labcorp) [2] or the Lipofraction NMR test (Cleveland Clinic HeartLab); (ii) density gradient ultracentrifugation, such as the Vertical Auto Profile (VAP) II test (Atherotec, AL); and (iii) ion mobility tests, such as the Cardio IQ® test (Quest Diagnostics). Ion mobility lipoprotein fractionation is the newest of the 3 methods and provides direct measurement of lipid subclasses including apolipoprotein B (Apo B), and Lp(a). It uses gas-phase electrophoresis to separate lipoproteins by size, which are directly counted by light scattering as they exit a separation chamber [3]. Validation studies demonstrated assay coefficients of variation of <1% for LDL particle size, <18% for HDL, and <16% for LDL fractions [4]. In contrast, NMR assays estimate lipoprotein concentration indirectly using methyl group signals, with correlation coefficients of 0.98 to 0.997 and precision of < 3.7% [5, 6]. Lastly, the VAP II method uses vertical-spin density-gradient ultracentrifugation to separate and directly quantify lipoprotein subclasses by spectrophotometric absorbance curves [7]. VAP II measurements of HDL cholesterol correlating with gel electrophoresis (*r* = 0.861, variation of 5%-9%) [8]. All 3 lipoprotein fractionation methods are reproducible, but we used ion mobility (Cardio IQ®) due to its availability and ability to measure particles without interference from stains or sample modifications.

While standard lipid guidelines focus on total cholesterol, LDL-C, HDL-cholesterol (HDL-C), and triglycerides, lipid subclasses are rarely measured and lack clear management guidelines. This case tracks lipid subclasses measured by ion mobility fractionation hyperlipidemic patient treated with a proprotein convertase subtilisin/kexin type 9 inhibitor (PCSK9i) and bempedoic acid.

## Case Presentation

A 54-year-old female individual with a history of hyperlipidemia, hypothyroidism, latent autoimmune diabetes of the adult (LADA) with hypoglycemia unawareness, vitiligo, gastroparesis probably secondary to endometriosis surgery, and asthma presented with exercise-limiting severe acute pleuritic chest pain and dyspnea. The patient had a past medical history of painful endometriosis resulting in laparoscopic abdominal surgery. She reported having undergone menopause in her late 40s. Type 2 diabetes had been diagnosed 2 years prior and shortly after found to have been LADA. Early after her diagnosis, she developed hypoglycemia unawareness due to the high frequency of lows she experienced during and after exercise while on multiple daily insulin injection therapy. At presentation, her body mass index was 20.43 kg/m^2^ (weight 119 lbs [56.7 kg]; height 5 feet 4.25 inches [163.2 cm]), which she reported was above her baseline weight of 108 lbs (49 kg). Prior to presentation, she reported being highly active— engaging in scuba diving, free diving, bodyboarding, surfing, and canoeing every day. On examination, she did not present with physical findings typical of familial hyperlipidemia (xanthomas or corneal arcus). However, she had a family history of premature myocardial infarction in her mother (mid 50s) and known hyperlipidemia. She underwent a full pulmonary and cardiac workup locally and at Mayo Clinic, which were negative for pulmonary embolism or cardiac ischemia.

## Diagnostic Assessment

The patient presented with an elevated LDL (226 mg/dL [5.85 mmol/L]) in December 2023 since stopping her rigorous physical exercise regimen due to her symptoms, which began in September 2023. Locally, she presented with chest pain, and the emergency department workup showed negative cardiac biomarkers and normal chest x-ray, although her V/Q scan was abnormal. Further evaluation revealed a normal exercise stress myocardial perfusion imaging study. While mild carotid artery stenosis and mild aortic calcification were noted on computerized tomography (CT) scan, there was no evidence of CAD on CT angiogram (coronary score: 0) or evidence of myocardial ischemia on exercise echocardiogram. A VO2 exercise test performed at Mayo Clinic was unrevealing with normal exercise parameters, and no clear pulmonary or cardiac limitations. Similarly, she had a normal cardiac positron emission tomography (PET) scan with no evidence of abnormal microvascular flow or ischemia. She was evaluated by other specialists and no other causes for her symptoms were found.

Her standard lipid panel in January 2024 were as follows: cholesterol 234 mg/dL (reference range < 200 mg/dL [6.05 mmol/L, reference range < 5.17 mmol/L]); HDL 51 mg/dL (reference range > 40 mg/dL [1.32 mmol/L, reference range >1.03 mmol/L]); triglycerides 104 mg/dL (reference range < 150 mg/dL [1.17 mmol/L, reference range < 1.69 mmol/L]); LDL-C 161 mg/dL (reference range < 100 mg/dL [4.17 mmol/L, reference range <2.59 mmol/L]). Her Cardiac IQ® panel in January 2024 revealed an abnormal lipid particle size distribution (HDL large: 5797 nmol/L [optimal: > 6729 nmol/L], LDL medium: 608 nmol/L [optimal: < 215 nmol/L], LDL particle number: 1962 nmol/L [optimal: < 1138 nmol/L], LDL peak size: 217.4 Angstrom [optimal: > 222.9 Angstrom], LDL small: 381 nmol/L [optimal: < 142 nmol/L], and Lp(a): 88 nmol/L [optimal: < 75 nmol/L]), with predominantly sdLDL particles, consistent with a high-risk atherogenic profile [8]. Additionally, she had high levels of Apo B (129 mg/dL [optimal: < 90 mg/dL]), an independent risk factor for CAD [9] (Table 1).

**Table 1.** Patient's standard lipid panel and lipid fractionation assay laboratory results

		Start ezetimibe		Chest pain & dyspnea began 9/2023		Start evolocumab (PCSK9i)	Start bempedoic acid			
Lab value	5/14/22	8/6/22	12/30/22	4/1/23	12/9/23	1/16/24	5/3/24	8/10/24	11/29/24	2/6/25
**Total cholesterol**	221 mg/dL (5.72 mmol/L)	215 mg/dL (5.57 mmol/L)	194 mg/dL (5.02 mmol/L)	203 mg/dL (5.26 mmol/L)	340 mg/dL (8.81 mmol/L)	234 mg/dL (6.06 mmol/L)	200 mg/dL (5.18 mmol/L)	132 mg/dL (3.42 mmol/L)	145 mg/dL (3.76 mmol/L)	131 mg/dL (3.39 mmol/L)
**Triglycerides**	92 mg/dL (1.04 mmol/L)	82 mg/dL (0.93 mmol/L)	127 mg/dL (1.44 mmol/L)	80 mg/dL (0.9 mmol/L)	70 mg/dL (0.79 mmol/L)	104 mg/dL (1.18 mmol/L)	60 mg/dL (0.68 mmol/L)	50 mg/dL (0.56 mmol/L)	61 mg/dL (0.69 mmol/L)	45 mg/dL (0.51 mmol/L)
**HDL-C**	62 mg/dL (1.61 mmol/L)	64 mg/dL (1.66 mmol/L)	66 mg/dL (1.71 mmol/L)	88 mg/dL (2.28 mmol/L)	100 mg/dL (2.59 mmol/L)	51 mg/dL (1.32 mmol/L)	79 mg/dL (2.05 mmol/L)	81 mg/dL (2.1 mmol/L)	81 mg/dL (2.1 mmol/L)	84 mg/dL (2.18 mmol/L)
**HDL large** Ref range: 0.1948- 0.6916 mg/dL) 5038-17,886 nmol/L Optimal: >0.2602 (>6729 nmol/L)	ND	ND	ND	ND	ND	0.2241 mg/dL (5797 nmol/L)	0.3238 mg/dL (8375 nmol/L)	ND	0.2889 mg/dL (7471 nmol/L)	0.3337 mg/dL (8631 nmol/L)
**Non-HDL cholesterol**	ND	ND	128 mg/dL (3.32 mmol/L)	115 mg/dL (2.98 mmol/L)	240 mg/dL (6.22 mmol/L)	183 mg/dL (4.74 mmol/L)	121 mg/dL (3.13 mmol/L)	51 mg/dL (1.32 mmol/L)	64 mg/dL (1.66 mmol/L)	47 mg/dL (1.22 mmol/L)
**LDL-C**	141 mg/dL (3.65 mmol/L)	135 mg/dL (3.5 mmol/L)	103 mg/dL (2.67 mmol/L)	99 mg/dL (2.56 mmol/L)	226 mg/dL (5.85 mmol/L)	161 mg/dL (4.17 mmol/L)	109 mg/dL (2.82 mmol/L)	41 mg/dL (1.06 mmol/L)	50 mg/dL (1.29 mmol/L)	34 mg/dL (0.88 mmol/L)
**LDL small** Ref range: 0.0044-0.0149 mg/dL (115-386 nmol/L) Optimal: <0.0055 mg/dL (<142 nmol/L)	ND	ND	ND	ND	ND	0.0147 mg/dL (381 nmol/L)	0.006 mg/dL (156 nmol/L)	ND	0.0036 mg/dL (93 nmol/L)	0.0032 mg/dL (83 nmol/L)
**LDL medium** Ref range: 0.0047- 0.0154 mg/dL (121-397 nmol/L) Optimal: <0.0083 mg/dL (<215 nmol/L)	ND	ND	ND	ND	ND	0.0235 mg/dL (608 nmol/L)	0.0089 mg/dL (230 nmol/L)	ND	0.0041 mg/dL (107 nmol/L)	0.0027 mg/dL (69 nmol/L)
**LDL particle no.** Ref range: 1016-2185 nmol/L Optimal: < 1138 nmol/L	ND	ND	ND	ND	ND	1962 nmol/L	1346 nmol/L	ND	639 nmol/L	699 nmol/L
**LDL peak size** Ref range: 216-234.3 Angstroms (21.6-23.43 nm) Optimal: >222.9 Angstroms (>22.29 nm)	ND	ND	ND	ND	ND	217.4 Angstroms (21.74 nm)	226.1 Angstroms (22.61 nm)	ND	219.9 Angstroms (21.99 nm)	218.3 Angstroms (21.83 nm)
**LDL pattern**	ND	ND	ND	ND	ND	A	A	ND	A	A
**Lipoprotein (a)** Optimal: <313 mg/L (<75 nmol/L)	ND	ND	ND	ND	ND	367 mg/L (88 nmol/L)	563 mg/L (135 nmol/L)	ND	417 mg/L (100 nmol/L)	488 mg/L (117 nmol/L)
**Apo B**	ND	ND	ND	ND	ND	129 mg/dL (0.0025 mmol/L)	79 mg/dL (0.0015 mmol/L)	ND	44 mg/dL (0.0009 mmol/L)	32 mg/dL (0.0006 mmol/L)
**HbA1c**	ND	ND	7.5% (58 mmol/mol)	7.0% (53 mmol/mol)	7.0% (53 mmol/mol)	ND	7.2% (55 mmol/mol)	7.2% (55 mmol/mol)	7.2% (55 mmol/mol)	7.4% (57 mmol/mol)
**CGM 90-day target range (70-180 mg/dL, 3.89-9.99 mmol/L)**			76%	83%	87%	88%	94%	89%	84%	87%

Abbreviations: Apo B, apolipoprotein B; CGM, continuous glucose monitor; HbA1c, hemoglobin A1c; HDL, high-density lipoprotein; LDL, low-density lipoprotein; ND, no data.

## Treatment

Although there was no genetic testing available, because of her abnormal lipid studies (LDL > 225 mg/dL), family history, and clinical presentation we suspected a familial hyperlipidemic disorder, such as heterozygous familial hyperlipidemia. After her chest pain began in September 2023 and abnormal lipid fractionation studies were noted, a PCSK9i (evolocumab 140 mg/mL) was started. The patient was already on ezetimibe 10 mg prior to presentation due to statin intolerance (myalgias, nausea, vomiting, and diarrhea) with atorvastatin and rosuvastatin. Addition of evolocumab reduced her LDL-C from 161 to 109 mg/dL (4.16 to 2.82 mmol/L). Lipid fractionation studies showed a reduction in Apo B from 129 to 79 mg/dL (0.0025 to 0.0015 mmol/L) after about 4 months, with improved HDL large particle size from 5797 to 8375 nmol/L (0.2241 to 0.3238 mg/dL) and a decrease in LDL medium particle size from 608 nmol/L (0.0235 mg/dL) to 230 nmol/L (0.0089 mg/dL) and LDL particle number from 1962 to 1346 nmol/L. Levels of sdLDL decreased significantly from 381 nmol/L (0.0147 mg/dL) to 156 nmol/L (0.006 mg/dL). Paradoxically, Lp(a) worsened from 88 nmol/L (367 mg/L) to 135 nmol/L (563 mg/L). Bempedoic acid was added, which further reduced sdLDL particles to 93 nmol/L (0.0036 mg/dL) and Lp(a) to 100 nmol/L (417 mg/dL) after 6 months of therapy. For diabetes management she was switched from multiple daily insulin injection to a closed-loop insulin pump system with improved glycemic control (Table 1) and reduction of hypoglycemic episodes.

## Outcome and Follow-Up

Over the past 2 years of follow-up, she has been tolerating triple anti-hypercholesterolemic medications and has been resuming regular physical activity. She has reported improvement of her chest pain episodes and dyspnea—noting a decreased need for nitroglycerin therapy from 3 times per week to now on an as-needed basis. At her most recent follow-up visit in February 2025, she reported not needing nitroglycerin for the past month. Her total LDL-C at a nadir of 34 mg/dL (0.88 mmol/L) and lipid fractionation studies showed significant improvement in LDL particle number, sdLDL, and Apo B levels (Table 1).

## Discussion

This patient carried a high-risk profile for CVD—including a family history of myocardial infarction, early menopause, and diabetes. Her chest pain was concerning for a coronary etiology and improved with nitroglycerin despite no evidence of obstructive CAD or ischemia, suggesting possible microvascular disease or vasospastic angina. We treated the patient as if she had underlying CAD and intensified her lipid-lowering regimen, which lowered her non-HDL cholesterol from the standard lipid panel from 240 to 47 mg/dL (6.22 to 1.22 mmol/L), a dramatic drop from triple to double digits. This was accompanied by significant reductions in sdLDL and ApoB on lipid fractionation, although Lp(a) levels remained elevated. Since Lp(a) is largely genetically determined but can be influenced by estrogen and thyroid hormone fluctuations [10], we attributed the increase to hormonal changes.

The atherogenic mechanisms of lipoprotein subclasses—such as sdLDL—are not fully understood but may involve weaker binding affinities to hepatic LDL receptors delaying clearance, increased susceptibility to oxidation with subsequent foam cell formation, and endothelial dysfunction [11]. Studies have linked elevated sdLDL to increased CVD risk. For example, Otrante et al found that sdLDL levels were higher in acute coronary syndrome patients compared to healthy controls [12], and data from the Women's Health Study showed that sdLDL-C is strongly associated with increased risk for myocardial infarction [13]. However, more robust evidence is needed to warrant the use of sdLDL as a clinical biomarker in lieu of standard risk factor assessments, given its high cost, relative unavailability in traditional laboratories [8], and uncertain benefit from targeted reduction [14]. Further research may help elucidate the most appropriate treatment options for specific sets of aberrant ion mobility lipoprotein fractionation results.

Two randomized trials (FOURIER and SPIRE-2) have shown that PCSK9i reduced the risk of CVD, while the ODYSSEY randomized trial showed that PCSK9i reduced the risk of all-cause mortality in high-risk patients. High-risk patients are generally those with familial hypercholesterolemia, those who do not tolerate statin therapy, or those who continue to have high risk after treatment with statins [15]. However, there is insufficient evidence that LDL subfractions and LDL particle numbers will add predictive value to a standard lipid panel when determining whether to initiate PCSK9i therapy [15]. This is in part due to the lack of standardization in determining which LDL subfractions and LDL particle numbers to include in this determination as well as different units reported depending on the type of lipoprotein fractionation assay used [15]. The paucity of literature with fractionation assay methodologies made it difficult to compare her laboratory values to other similar cases. Bempedoic acid is an ATP citrate lyase inhibitor, which increases LDL receptor expression and thus plasma LDL clearance. This case supports the use of a PCSK9i plus bempedoic acid as an effective treatment in a patient with proposed familial hyperlipidemia with high-risk lipoprotein particles on ion mobility lipoprotein fractionation testing. Furthermore, this case highlights the importance of using lipid fractionation studies as a clinical tool for monitoring high-risk patients with hyperlipidemia—as it was only through this testing that the need for a PCSK9i was identified. Further studies are needed to investigate the efficacy of other lipid-lowering agents in larger cohorts of patients using lipid fractionation laboratory results to better formulate standardized treatment guidelines for patients at high risk for CVD.

## Learning Points

- Ion mobility lipoprotein fractionation studies have valuable efficacy in identifying patients who have abnormal standard lipid panels and are at high risk for CVD.
- This case demonstrates that sdLDL and LDL particle number may be useful as markers for increased CVD risk, which can be proactively improved through PCSK9i therapy before a cardiovascular event occurs.
- The lack of standardization in lipid fractionation assays makes it difficult to develop standardized treatment guidelines for patients at high risk for CVD.

## Data Availability

Original data generated and analyzed during this study are included in this published article.

## Abbreviations

Apo B: apolipoprotein B
CAD: coronary artery disease
CVD: cardiovascular disease
HDL-C: high-density lipoprotein cholesterol
LADA: latent autoimmune diabetes of the adult
LDL-C: low-density lipoprotein cholesterol
Lp(a): lipoprotein (a)
NMR: nuclear magnetic resonance
PCSK9i: proprotein convertase subtilisin/kexin type 9 inhibitor
sdLDL: small, dense low-density lipoprotein

## References

[1] Sacks FM, Campos H. Low-density lipoprotein size and cardiovascular disease: a reappraisal. J Clin Endocrinol Metab. 2003;88(10):4525‐4532. 14557416 10.1210/jc.2003-030636

[2] Emeasoba EU, Ibeson E, Nwosu I, Montemarano N, Shani J, Shetty VS. Clinical relevance of nuclear magnetic resonance LipoProfile. Front Nucl Med. 2022;2:960522. 39354981 10.3389/fnume.2022.960522 PMC11440956

[3] Krauss RM . Lipoprotein subfractions and cardiovascular disease risk. Curr Opin Lipidol. 2010;21(4):305‐311. 20531184 10.1097/MOL.0b013e32833b7756

[4] Caulfield MP, Li S, Lee G, et al Direct determination of lipoprotein particle sizes and concentrations by ion mobility analysis. Clin Chem. 2008;54(8):1307‐1316. 18515257 10.1373/clinchem.2007.100586

[5] Jiménez B, Holmes E, Heude C, et al Quantitative lipoprotein subclass and low molecular weight metabolite analysis in human Serum and plasma by ^1^H NMR spectroscopy in a multilaboratory trial. Anal Chem. 2018;90(20):11962‐11971. 30211542 10.1021/acs.analchem.8b02412

[6] Garcia E, Bennett DW, Connelly MA, et al The extended lipid panel assay: a clinically-deployed high-throughput nuclear magnetic resonance method for the simultaneous measurement of lipids and apolipoprotein B. Lipids Health Dis. 2020;19(1):247. 33261644 10.1186/s12944-020-01424-2 PMC7709389

[7] Kulkarni KR, Garber DW, Jones MK, Segrest JP. Identification and cholesterol quantification of low density lipoprotein subclasses in young adults by VAP-II methodology. J Lipid Res. 1995;36(11):2291‐2302. 8656067

[8] Stanciulescu LA, Scafa-Udriste A, Dorobantu M. Exploring the association between low-density lipoprotein subfractions and Major adverse cardiovascular outcomes-A comprehensive review. Int J Mol Sci. 2023;24(7):6669. 37047642 10.3390/ijms24076669 PMC10095470

[9] Sniderman AD, Thanassoulis G, Glavinovic T, et al Apolipoprotein B particles and cardiovascular disease: a narrative review. JAMA Cardiol. 2019;4(12):1287‐1295. 31642874 10.1001/jamacardio.2019.3780 PMC7369156

[10] Enkhmaa B, Berglund L. Non-genetic influences on lipoprotein(a) concentrations. Atherosclerosis. 2022;349:53‐62. 35606076 10.1016/j.atherosclerosis.2022.04.006 PMC9549811

[11] Chary A, Tohidi M, Hedayati M. Association of LDL-cholesterol subfractions with cardiovascular disorders: a systematic review. BMC Cardiovasc Disord. 2023;23(1):533. 37914996 10.1186/s12872-023-03578-0 PMC10621218

[12] Otrante A, Bounafaa A, Berrougui H, et al Small dense LDL level and LDL/HDL distribution in acute coronary syndrome patients. Biomedicines. 2023;11(4):1198. 37189816 10.3390/biomedicines11041198 PMC10135780

[13] Duran EK, Aday AW, Cook NR, Buring JE, Ridker PM, Pradhan AD. Triglyceride-rich lipoprotein cholesterol, small dense LDL cholesterol, and incident cardiovascular disease. J Am Coll Cardiol. 2020;75(17):2122‐2135. 32354380 10.1016/j.jacc.2020.02.059 PMC8064770

[14] Krauss RM . Small dense low-density lipoprotein particles: clinically relevant? Curr Opin Lipidol. 2022;33(3):160‐166. 35276699 10.1097/MOL.0000000000000824 PMC9197986

[15] Kjellmo CA, Hovland A, Lappegård KT. CVD risk stratification in the PCSK9 era: is there a role for LDL subfractions? Diseases. 2018;6(2):45. 29861477 10.3390/diseases6020045 PMC6023332

